# Microbial Enterotypes Shape the Divergence in Gut Fermentation, Host Metabolism, and Growth Rate of Young Goats

**DOI:** 10.1128/spectrum.04818-22

**Published:** 2023-01-10

**Authors:** Dangdang Wang, Guangfu Tang, Junjian Yu, Yuanyuan Li, Leiyu Feng, Huifeng Liu, Jiaxiao Li, Luyu Chen, Yangchun Cao, Junhu Yao

**Affiliations:** a College of Animal Science and Technology, Northwest A&F University, Yangling, Shaanxi, China; Jilin University

**Keywords:** enterotype, gut microbiome, co-occurrence network, growth rate, goat

## Abstract

Enterotypes can be useful tools for studying the gut microbial community landscape, which is thought to play a crucial role in animal performance. However, few studies have been carried out to identify enterotypes and their associations with growth performance in young goats. In this study, two enterotypes were categorized in 76 goats: cluster 1 (*n *=* *39) and cluster 2 (*n *=* *37). Compared to cluster 2, cluster 1 had greater growth rates, the concentrations of acetate, propionate, valerate, and total volatile fatty acids (VFA) in the gut. Several serum glycolipid metabolism parameters, including glucose, total cholesterol, high-density lipoprotein cholesterol (HDL-C), and low-density lipoprotein cholesterol (LDL-C), were also increased in cluster 1, while serum IgG was decreased in cluster 1. Using α-diversity analysis, we found a microbiome with lower richness and diversity in cluster 1. Some gut bacteria, including *Succinivibrio* and several members of the *Prevotellaceae* family, were enriched in cluster 1, while *Christensenellaceae R-7 group*, *Romboutsia*, and *Clostridium sensu stricto 1* were enriched in cluster 2. A co-occurrence network analysis revealed that the differential interaction patterns existed in two enterotypes, and microbial function prediction suggested that some nutrient metabolism-related pathways, including amino acid biosynthesis and starch and sucrose metabolism, were enriched in cluster 1. Furthermore, a correlation analysis showed that enterotype-related bacteria were closely correlated with gut fermentation, serum biochemistry, and growth rate. Overall, our data provide a new perspective for understanding enterotype characteristics in goats, offering insights into important microbial interaction mechanisms for improving the growth performance of ruminant animals.

**IMPORTANCE** The intricate relationships between a host animal and its resident gut microbiomes provide opportunities for dealing with energy efficiency and production challenges in the livestock industry. Here, we applied the enterotype concept to the gut microbiome in young goats and found that it can be classified into two enterotypes which are apparently associated with divergences in gut fermentation, blood biochemistry, and goat growth rates. The microbial co-occurrence networks and function predictions differed between the two enterotypes, suggesting that the formation of host phenotype may be modified by different bacterial features and complex bacterial interactions. The characteristics of enterotypes related to growth performance in young goats may enable us to improve long-term production performance in goat industry by modulating the gut microbiome during early life.

## INTRODUCTION

Trillions of microbes rapidly colonize the gastrointestinal tract during birth and afterwards, and they maintain a delicate balance in a symbiotic relationship with the host (1). Previous studies have proven that the early-life gut microbiota plays fundamental roles in animal health and performance by providing nutrients (2), regulating the immune system (3), promoting tissue maturation (4), and preventing pathogen colonization (5). Moreover, a variety of interventions can be used during the early life to manipulate the gut microbiota, which may have long-term impacts on the host (6, 7). Specifically, changes in the gut microbiota induced by fecal microbiota transplantation contributed to alleviating diarrhea in pre-weaning calves and further exerted positive effects in promoting the systemic metabolome and growth performance of adult cattle (8). Therefore, an increased fundamental understanding of the gut microbiome of young ruminants is essential to develop novel approaches for improving livestock growth and production.

The structure of the gut microbiota varies greatly among individuals, making it possible to classify different individuals into several enterotypes (9). The essence of enterotypes is stratifying the global gut microbiome into several categories using a dimension reduction algorithm; these serve as “highly populated areas in a multidimensional space of community composition” (9). Recent studies have proven that enterotypes can be a useful tool for studying the gut microbial community landscape and its influences on the host (10, 11). In ruminants, one recent study in dairy cows reported that milk yield and body weight were significantly higher in unclassified *Spirochaetaceae* enterotype cows than in *Bifidobacterium* enterotype cows (12). It is also noteworthy that yaks shifted their enterotypes in response to dietary changes between the warm and cold seasons to improve nitrogen and energy utilization (13). To our knowledge, the concept of enterotypes has been seldom used in young goats, and limited research has been carried out to identify the functions of enterotypes and their effects on growth rates in young goats.

We hypothesized different enterotypes exist in the gut and that they may have indispensable influences in regulating gut fermentation and host metabolism, thus affecting growth rates in young goats. Therefore, in this study, we performed enterotype analysis for 76 young goats which were fed the same diet and raised under the same conditions. We analyzed the relationships of enterotypes with gut fermentation, blood biochemistry, and growth rates in goats.

## RESULTS

### Enterotype clustering of gut microbiome in young goats.

**FIG 1:**
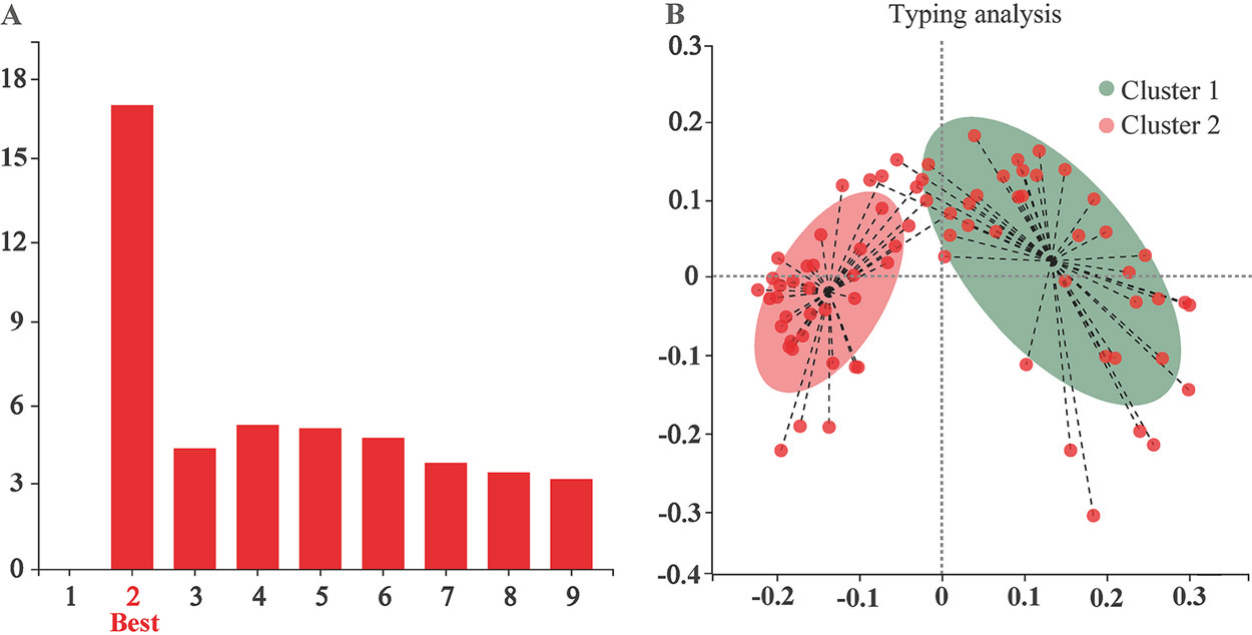
Gut microbial clustering in young goats (*n *=* *76). (A) Optimal number of gut enterotypes separation. The *x* axis shows the number of enterotypes; the *y* axis shows Calinski-Harabasz index. (B) Principal coordinate analysis (PCoA) plot of gut enterotypes based on Jensen-Shannon distance.

The results of enterotype analysis on the gut microbiome in young goats (*n *=* *76) are shown in Fig. 1. The microbial communities of all goats were stratified into two robust enterotypes with the strongest support by the maximum Calinski-Harabasz index (Fig. 1A). Both cluster 1 (*n *=* *39) and cluster 2 (*n *=* *37) were driven by ASV2354, which belongs to *Turicibacter* (Fig. 1B). In this research, all goats were grouped according to cluster 1 and cluster 2.

### The growth rate, gut fermentation, and serum parameters.

**FIG 2:**
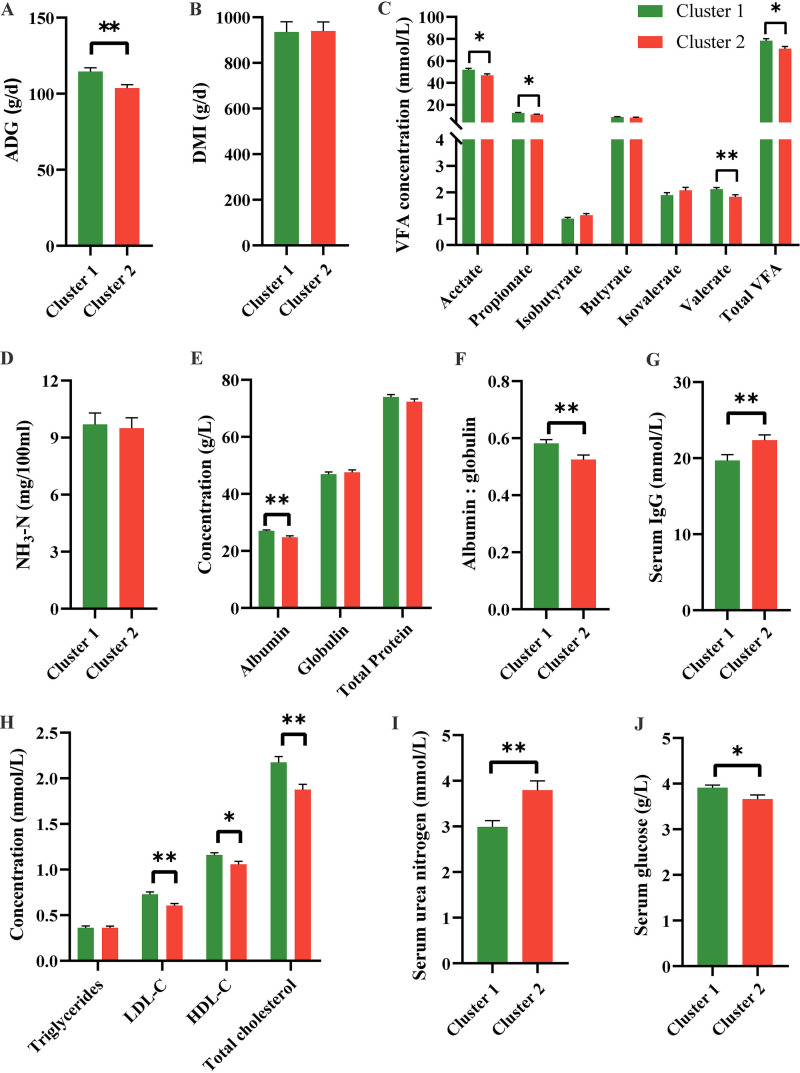
Growth performance, gut fermentation and serum parameters in young goats with different enterotypes. (A) Average daily gain (ADG). (B) Dry matter intake (DMI). (C) Fecal volatile fatty acids (VFA). (D) Fecal ammonia nitrogen (NH_3_-N). (E) Serum albumin, globulin and total protein. (F) The ratio of albumin to globulin. (G) Serum IgG. (H) Serum triglycerides, low-density lipoprotein cholesterol (LDL-C), high-density lipoprotein cholesterol (HDL-C) and total cholesterol. (I) Serum urea nitrogen. (J) Serum glucose. Bars represent standard error (SE). *, *P < *0.05; **, *P < *0.01.

The average daily gain (ADG), dry matter intake (DMI), gut fermentation, and several serum parameters in clusters 1 and 2 were further explored. We found that the ADG was greater in cluster 1 than in cluster 2 (*P = *0.002, Fig. 2A), but the DMI did not differ between groups (*P = *0.375, Fig. 2B). The concentrations of acetate, propionate, valerate and total volatile fatty acids (VFA) were greater in cluster 1 than in cluster 2 (*P < *0.05, Fig. 2C). There were no differences in serum globulin (Glob), total protein (TP) and triglycerides, or fecal ammonia nitrogen (NH_3_-N) between the two enterotypes (*P > *0.05, Fig. 2D, E, and H). However, compared to cluster 2, cluster 1 had higher concentrations of albumin (Alb), total cholesterol, high-density lipoprotein cholesterol (HDL-C), low-density lipoprotein cholesterol (LDL-C), glucose, and the ratio of Alb to Glob, but lower concentrations of IgG and urea nitrogen in serum (*P < *0.05, Fig. 2E to J). These results suggest that enterotypes have important effects on gut fermentation, host metabolism, and growth rates in young goats.

### The gut microbial community structures and compositions.

**FIG 3:**
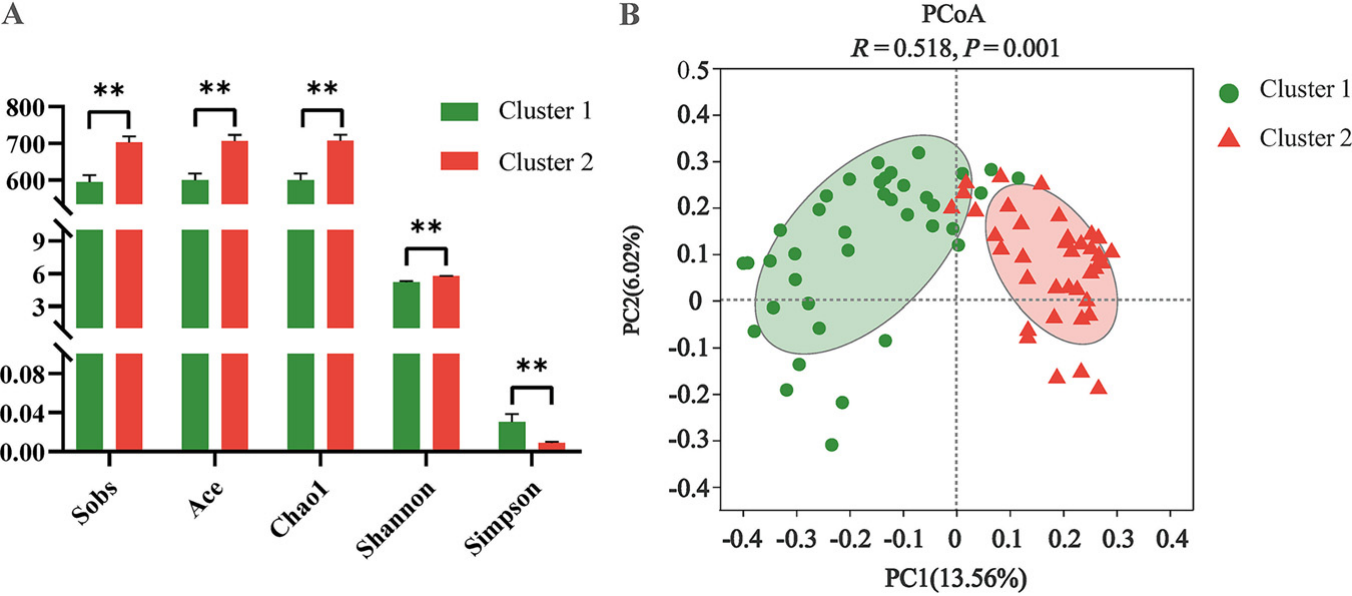
Microbial α-diversity and β-diversity in young goats with different enterotypes. (A) α-Diversity. (B) PCoA plot of the fecal microbiome based on Bray-Curtis distance matrix. Bars represent SE. *, *P < *0.05; **, *P < *0.01.

The Sobs, Ace, Chao1, and Shannon indices were lower in cluster 1, while the Simpson index was higher in cluster 1 than in cluster 2 (*P < *0.01, Fig. 3A). Principal-coordinate analysis (PCoA) based on the Bray-Curtis distance showed that there was a significant difference in microbial community structure between the two enterotypes (*P = *0.001, Fig. 3B).

The taxonomic differences between the two enterotypes were analyzed at the family and genus levels. Among the top 15 families, the relative abundances of *Ruminococcaceae*, *Prevotellaceae*, *Muribaculaceae*, and *Succinivibrionaceae* were increased in cluster 1; while the relative abundances of *Rikenellaceae*, *Christensenellaceae*, and *Bacteroidaceae* were decreased in cluster 1 compared to cluster 2 (*P < *0.05, Table 1).

**TABLE 1:** Differential microbiota analysis of gut microbiota in two enterotypes at the family level (%, top 15)^*a*^

Taxon	Cluster 1	Cluster 2	*P*
*Oscillospiraceae*	15.56 ± 0.59	15.33 ± 0.76	0.217
*Lachnospiraceae*	11.78 ± 0.76	13.60 ± 0.87	0.052
*Rikenellaceae*	10.80 ± 0.57	6.30 ± 0.62	<0.001
*Christensenellaceae*	8.28 ± 0.43	3.19 ± 0.31	<0.001
*Spirochaetaceae*	5.32 ± 0.61	4.93 ± 0.60	0.954
*Bacteroidaceae*	4.82 ± 0.32	2.49 ± 0.25	<0.001
*Ruminococcaceae*	3.94 ± 0.24	5.96 ± 0.52	0.001
*Prevotellaceae*	3.80 ± 0.40	9.37 ± 1.18	<0.001
Oscillospirales_f_UCG-010	3.50 ± 0.28	2.80 ± 0.24	0.080
Bacteroidales_RF16_group	2.47 ± 0.30	2.02 ± 0.26	0.365
Eubacterium_coprostanoligenes_group	2.14 ± 0.15	1.88 ± 0.14	0.391
*Erysipelotrichaceae*	2.05 ± 0.34	3.63 ± 1.30	0.319
Norank_o_Clostridia_UCG-014	1.96 ± 0.15	2.74 ± 0.30	0.052
*Muribaculaceae*	1.78 ± 0.24	4.78 ± 0.87	<0.001
*Succinivibrionaceae*	1.65 ± 0.45	4.32 ± 1.39	0.016

a Data are shown as mean ± standard error. Cluster 1 (*n* = 39), cluster 2 (*n* = 37).

Of the genera with a relative abundance greater than 1%, *norank Muribaculaceae*, *Succinivibrio*, *Prevotella*, *Roseburia*, *Prevotellaceae UCG-003*, and *Prevotellaceae NK3B31 group* were enriched in cluster 1, while *Rikenellaceae RC9 gut group*, *Christensenellaceae R-7 group*, *Bacteroides*, *Oscillospiraceae NK4A214 group*, *norank F082*, *Romboutsia*, *Monoglobus*, and *Clostridium sensu stricto 1* were decreased in cluster 1 compared to cluster 2 (*P < *0.05, Table 2).

**TABLE 2:** Differential microbiota analysis of gut microbiota in two enterotypes at genus level (%, relative abundance > 1%)^*a*^

Taxon	Cluster 1	Cluster 2	*P*
Oscillospiraceae_UCG-005	10.82 ± 0.62	10.38 ± 0.49	0.941
unclassified_f_Lachnospiraceae	7.84 ± 0.62	8.62 ± 0.60	0.494
Rikenellaceae_RC9_gut_group	4.18 ± 0.50	7.96 ± 0.47	<0.001
Christensenellaceae_R-7_group	3.17 ± 0.30	8.23 ± 0.44	<0.001
Treponema	4.93 ± 0.59	5.31 ± 0.63	0.966
*Bacteroides*	2.49 ± 0.24	4.82 ± 0.33	<0.001
Norank_f_Muribaculaceae	4.72 ± 0.83	1.78 ± 0.24	<0.001
Norank_f_UCG-010	2.80 ± 0.24	3.50 ± 0.28	0.080
*Turicibacter*	3.53 ± 1.27	1.99 ± 0.35	0.213
*Succinivibrio*	3.84 ± 1.33	1.60 ± 0.46	0.030
Norank_f_norank_o_Clostridia_UCG-014	2.74 ± 0.29	1.96 ± 0.15	0.052
*Ruminococcus*	2.07 ± 0.21	2.45 ± 0.22	0.148
Norank_f_Bacteroidales_RF16_group	2.02 ± 0.26	2.47 ± 0.31	0.365
*Alistipes*	1.96 ± 0.18	2.43 ± 0.20	0.068
*Prevotella*	3.32 ± 0.66	0.93 ± 0.20	<0.001
Norank_f_Eubacterium_coprostanoligenes_group	1.88 ± 0.14	2.14 ± 0.16	0.391
Oscillospiraceae_UCG-002	1.69 ± 0.19	1.70 ± 0.14	0.468
Oscillospiraceae_NK4A214_group	1.02 ± 0.07	1.78 ± 0.12	<0.001
*Phascolarctobacterium*	1.65 ± 0.25	1.21 ± 0.11	0.376
Norank_f_F082	0.84 ± 0.19	1.88 ± 0.28	<0.001
*Romboutsia*	1.15 ± 0.22	1.44 ± 0.21	0.042
*Monoglobus*	1.05 ± 0.11	1.51 ± 0.10	<0.001
*Roseburia*	1.91 ± 0.47	0.52 ± 0.08	<0.001
Prevotellaceae_UCG-003	1.75 ± 0.45	0.78 ± 0.13	0.029
Clostridium_sensu_stricto_1	0.95 ± 0.18	1.37 ± 0.20	0.017
Norank_f_Ruminococcaceae	1.54 ± 0.36	0.76 ± 0.06	0.111
Prevotellaceae_NK3B31_group	2.08 ± 0.39	0.16 ± 0.07	<0.00

a Data are shown as mean ± standard error. Cluster 1 (*n* = 39), cluster 2 (*n* = 37).

### Bacterial-bacterial interactions of co-occurrence networks.

Because bacterial-bacterial interactions are key modulators in shaping the gut microbial community, we further investigated whether there were differences in bacterial-bacterial interactions between enterotypes; bacterial co-occurrence networks were constructed at the genus level for clusters 1 and 2. Information for all differential genera which were used in co-occurrence networks is presented in Table S2.

Interestingly, we found that the co-occurrence and co-exclusion patterns were different between the two enterotypes. In the co-occurrence network of cluster 1, there were positive associations among cluster 1-enriched genera and among cluster 2-enriched genera, while multiple negative associations were found between cluster 1-enriched genera and cluster 2-enriched genera (Spearman’s correlation, *P < *0.05, Fig. S1A). However, these bacterial interactions become ambiguous in the network of cluster 2. (Fig. S1B).

**FIG 4:**
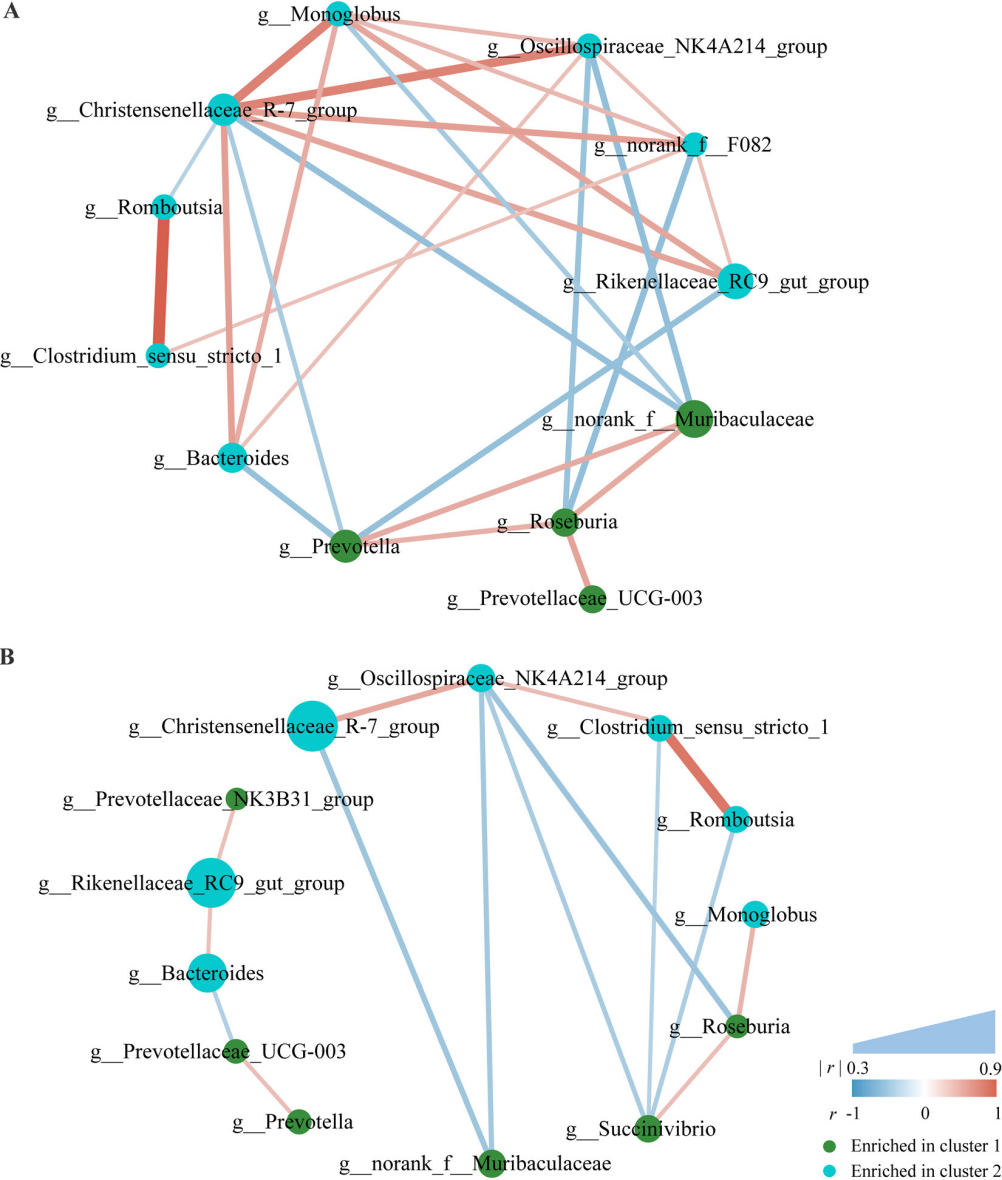
Co-occurrence network of differential microbial genera (relative abundance > 1%) in young goats with different enterotypes. (A) Co-occurrence network of differential microbial genera in cluster 1. (B) Co-occurrence network of differential microbial genera in cluster 2. Red lines represent positive correlations, blue lines represent negative correlations. Line thickness represents strength of relatedness. Node colors indicate clusters in which the genera were enriched.

In particular, in the co-occurrence network of cluster 1, some genera which were enriched in cluster 2, including *Rikenellaceae RC9 gut group*, *Christensenellaceae R-7 group*, *Bacteroides*, *Oscillospiraceae NK4A214 group*, *norank F082*, and *Monoglobus*, positively correlated with each other. Three genera which were enriched in cluster 1, including *norank Muribaculaceae*, *Prevotella*, and *Roseburia*, positively correlated with each other and had multiple negative correlations with the cluster 2-enriched genera mentioned above (Fig. 4A). However, some genera, such as *Rikenellaceae RC9 gut group*, *Christensenellaceae R-7 group*, *Bacteroides*, *norank Muribaculaceae*, and *Prevotella*, lost their correlations with other genera in part or all in the co-occurrence network of cluster 2 (Fig. 4B).

### Microbial function prediction.

Various functional features were predicted from the 16S rRNA gene sequences based on the Kyoto Encyclopedia of Genes and Genomes (KEGG) database (Table S3). Of the top 15 KEGG pathways (level 3), 9 pathways, including amino sugar and nucleotide sugar metabolism, alanine, aspartate and glutamate metabolism, starch and sucrose metabolism, methane metabolism, amino acid biosynthesis, peptidoglycan biosynthesis, and phenylalanine, tyrosine, and tryptophan biosynthesis, were enriched in cluster 1 (*P < *0.05, Table 3). Meanwhile, 6 pathways, including microbial metabolism in diverse environments, carbon metabolism, carbon fixation pathways in prokaryotes, and glycine, serine and threonine metabolism, were enriched in cluster 2 (*P < *0.05, Table 3).

**TABLE 3:** Microbial function prediction in clusters 1 and 2 (%, top 15)^*a*^

KEGG pathway	Cluster 1	Cluster 2	*P*
Biosynthesis of secondary metabolites	9.189 ± 0.013	9.137 ± 0.015	0.002
Biosynthesis of amino acids	4.470 ± 0.022	4.416 ± 0.016	0.015
Microbial metabolism in diverse environments	4.268 ± 0.009	4.326 ± 0.010	<0.001
Carbon metabolism	2.809 ± 0.009	2.849 ± 0.008	0.001
ABC transporters	2.164 ± 0.027	2.085 ± 0.027	0.013
Amino sugar and nucleotide sugar metabolism	1.132 ± 0.009	1.099 ± 0.004	0.014
Oxidative phosphorylation	1.005 ± 0.008	1.035 ± 0.006	0.001
Carbon fixation pathways in prokaryotes	0.980 ± 0.007	1.006 ± 0.006	0.001
Alanine, aspartate and glutamate metabolism	0.975 ± 0.005	0.953 ± 0.003	<0.001
Glycine, serine and threonine metabolism	0.973 ± 0.004	0.981 ± 0.003	0.024
Starch and sucrose metabolism	0.962 ± 0.015	0.905 ± 0.008	0.001
Peptidoglycan biosynthesis	0.945 ± 0.006	0.924 ± 0.003	0.010
Phenylalanine, tyrosine and tryptophan biosynthesis	0.856 ± 0.004	0.845 ± 0.004	0.019
Mismatch repair	0.847 ± 0.005	0.851 ± 0.003	0.008
Methane metabolism	0.739 ± 0.003	0.725 ± 0.002	<0.001

a Data are shown as mean ± standard error. Cluster 1 (*n* = 39), cluster 2 (*n* = 37).

### Correlation analysis of gut bacteria with gut fermentation, serum parameters, and growth rate.

**FIG 5:**
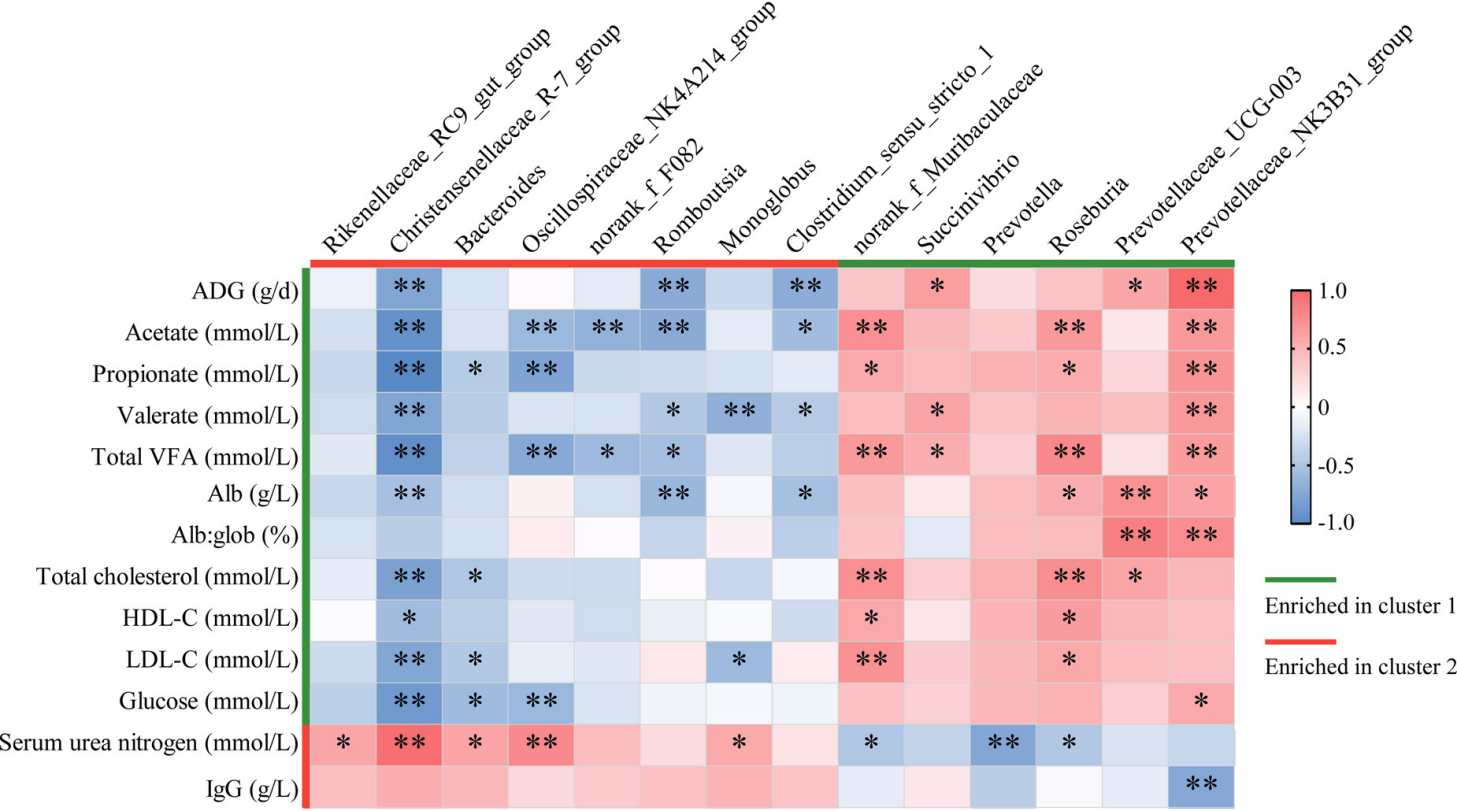
Association analysis of differential genera (relative abundance > 1%) with growth rates, gut fermentation, and serum biochemicals in young goats. *, *P < *0.05; **, *P < *0.01.

To explore the relationships of gut microbiota with gut fermentation, serum parameters, and growth rates, 14 differential genera (relative abundance > 1%) in the two enterotypes were selected for correlation analysis. As shown in Fig. 5, different microbe-trait interaction patterns were found between genera which were enriched in cluster 1 and those which were enriched in cluster 2.

Of the 6 genera which were enriched in cluster 1, 3 (*Succinivibrio*, *Prevotellaceae UCG-003*, and *Prevotellaceae NK3B31 group*) had significant positive correlations with ADG (*r *>* *0.25, *P < *0.05). The relative abundances of *norank Muribaculaceae*, *Roseburia*, and *Prevotellaceae NK3B31 group* were positively correlated with the concentrations of gut total VFA and its two major components, acetate and propionate (*r *>* *0.24, *P < *0.05). Two *Prevotellaceae* members, *Prevotellaceae UCG-003* and *Prevotellaceae NK3B31 group*, showed positive correlations with Alb and the ratio of Alb to Glob, and *Prevotellaceae NK3B31 group* negatively correlated with IgG (|*r|* > 0.26, *P < *0.05). The relative abundances of *norank Muribaculaceae* and *Roseburia* showed positive correlations with some serum parameters, such as total cholesterol, HDL-C, and LDL-C, while they were negatively correlated with serum urea nitrogen (|*r|* > 0.24, *P < *0.05).

Of the 8 genera which were enriched in cluster 2, 3 (*Christensenellaceae R-7 group*, *Romboutsia*, and *Clostridium sensu stricto 1*) had significant negative correlations with ADG (*r* < −0.33, *P < *0.01). Five genera, including *Christensenellaceae R-7 group*, *Oscillospiraceae NK4A214 group*, *norank F082*, *Romboutsia*, and *Clostridium sensu stricto 1* were negatively correlated with the concentrations of gut total VFA, acetate, and propionate in part or all (*r* < -0.28, *P < *0.05). *Christensenellaceae R-7 group*, *Bacteroides*, and *Oscillospiraceae NK4A214 group* had negative correlations with total cholesterol, HDL-C, LDL-C, and glucose in part or all (*r* < −0.24, *P < *0.05); while all of them positively correlated with serum urea nitrogen (*r *>* *0.26, *P < *0.05).

## DISSCUSION

Huge variations in the gut microbiota exist among individuals in time and space, which have been considered a barrier to determining the complex biological relationships between a host and its gut microbiome (9). As an effective analytical method, enterotyping provides a pioneering framework for understanding microbial variation in host metabolism and health (10, 11). However, to our knowledge, few studies have explored the relationship between enterotype and growth performance in goats. In this study, two enterotypes characterized by different community structures, microbial interactions, and microbial functions were identified in 76 young goats. We showed that enterotypes can affect gut fermentation, host metabolism, and growth performance in goats.

Compared to cluster 2, cluster 1 had increases in gut VFA and several serum metabolic parameters, including glucose, total cholesterol, HDL-C and LDL-C, which contributed to higher growth rate in cluster 1. We also noticed that serum urea nitrogen was decreased in cluster 1 compared to that in cluster 2. It has been reported that serum urea nitrogen concentrations can indicate the efficiency of protein utilization (14). Elevated serum urea nitrogen concentration can be caused by inefficient gut ammonia incorporation into microbial protein and hepatic deamination of amino acids mobilized from skeletal muscle (15, 16). Meanwhile, there was no difference in fecal NH_3_-N concentration between clusters 1 and 2, but several amino acid biosynthesis-related pathways were enriched in cluster 1, which may further indicate that more nitrogen is used to synthesize microbial protein in the gut. These traits variations may be influenced by microbiota diversity, composition, and bacterial interactions in different enterotypes.

Interestingly, a microbiome with lower richness and diversity was found in cluster 1 as shown by α-diversity analysis; this is in line with the view that efficient microbiomes are less complex but more specialized to support the host’s energy requirements (17, 18). In addition, the major bacteria that were enriched in cluster 1 were closely associated with gut VFA, serum metabolites, and growth rates. Of these, genera in the *Prevotellaceae* family generally possess extensive repertoires of polysaccharide utilization loci and carbohydrate-active enzymes targeting various plant polysaccharides, which could contribute to powerful VFA fermentation in the gut (19, 20). Recent genomic and culturomic studies have found that *Muribaculaceae* is functionally adept at complex carbohydrate degradation (21, 22). *Succinivibrio* is efficient in fermenting carbohydrate through the production of acetate and succinate, a precursor of propionate, as well as in metabolizing various fatty acids (23, 24). Several studies have reported that increased *Succinivibrio* may be beneficial to improve energy utilization efficiency and meet the energetic needs of animals (25, 26). Overall, the bacteria which were enriched in cluster 1 seem to have strong capability for carbohydrate fermentation and improving energy supply in young goats.

Conversely, some bacteria reported to be negatively correlated with animal health and growth performance were enriched in cluster 2. Ma et al. (27) reported that the relative abundances of *Rikenellaceae RC9 gut group* were higher in the ileum of growth-retarded yaks than in that of growth-normal yaks. *Christensenellaceae* is the most heritable taxon in the human gut microbiome and has a negative correlation with host body weight (28). One study in pigs also found that the *Christensenellaceae R-7 group* has a negative correlation with the ratio of goblet cell to villus height, indicating that it may play an important role in influencing ileal morphology (29). *Romboutsia* is a widely reported potential pathogen, and its relative abundance increases in various diseases, such as irritable bowel syndrome (30) and gastric cancer (31). Regarding *Clostridium sensu stricto 1*, evidence has shown that is elevated in the gut of lambs with diarrhea (32). In addition, Wang et al. (33) found that the enrichment of *Clostridium sensu stricto 1* in the sheep colon was positively related to some inflammatory cytokines and may be responsible for colonic mucosal damage and colonic dysfunction. Therefore, our current data revealed that gut microbial diversity and the composition of different enterotypes affect gut fermentation, and thus may have further effects on host metabolism and growth performance in young goats.

We also found that serum IgG was decreased in cluster 1 compared to that in cluster 2. As the most abundant serum immunoglobulin, IgG is conventionally linked to systemic infection because its induction requires the crossing of the barrier by antigens (34–36). High IgG immune responses generally occur during some specific disease status, such as in inflammatory bowel disease, in which the intestinal barrier is disrupted and pathogenic microorganisms translocate from the lumen (37–39). Low serum IgG may be related to the increased VFA-producing bacteria and gut VFA production in cluster 1, as VFA has positive effects on intestinal tissue development, epithelial barrier repair, and immune regulation, which is very beneficial for intestinal health (40). These results may indicate that cluster 1 had better intestinal homeostasis and fewer perturbations from potential pathogens.

Microorganisms do not exist in isolation but form complex ecological interaction communities, in which various interaction types, including cooperation, competition, and exploitation, exist among interaction partners (41). Interestingly, our co-occurrence network analysis revealed that the co-occurrence and co-exclusion patterns of gut microbiota were different between the two enterotypes. In cluster 1, the negative correlations between bacteria enriched in cluster 1 and those enriched in cluster 2 were more noticeable. Microbial interactions are major contributors to the establishment of microbial community states, and niche modification is one of the main mechanisms for this establishment (42). Proper competitive relationships between microorganisms are beneficial to increasing microbial community stability and have many positive effects on the host (43, 44). The different co-occurrence and co-exclusion patterns may indicate that the ecological niches were overwhelmingly occupied by taxa which favor gut fermentation, host metabolism, and host growth rates in cluster 1.

In summary, two enterotypes were found in young goats, and they were tightly associated with divergences in gut fermentation, host metabolism, and growth rates in goats. Some specific bacterial taxa and their associations with other bacteria may play important roles in these processes. Our results provide detailed and novel insights into the characteristics of enterotypes related to growth rate in young goats, which may enable us to improve animal performance in the goat industry by modulating the gut microbiome in early life. It is also noticeable that the rumen and its microbiota play particularly important roles in foodstuff degradation. Future studies should explore the characteristics of enterotypes in the rumen and their associations with hindgut enterotypes, which may deepen our knowledge of how the digestive tract microbiome features (diversity, structure, composition, and enterotype) affect ruminant feeding efficiency and performance.

## MATERIALS AND METHODS

### Animals, diets and sampling.

The experimental protocols and animal usage were approved by the Animal Use and Care Committee of Northwest A&F University (protocol no. NWAFAC1008).

A total of 76 6-month-old, healthy, female, Guan Zhong goat kids were used for the experiment. All animals were born in the same week and were weighed immediately after birth (mean ± standard error [SE], 3.11 ± 0.05 kg). Goat kids were fed the same diet and raised under the same conditions from birth until sampling. After birth, goat kids were raised by their dams for the first 3 days and then transferred to the lamb barn with mixed-milk feeding until they were weaned. The alfalfa hay and concentrate mixture were introduced to goat kids for supplementary feeding from 15 d old and 30 d old, respectively. All kids were weaned at 3 months of age. After weaning, a ration consisting of forage and concentrate (60:40) was provided *ad libitum* three times daily at 07:30, 13:00, and 19:00 h, and water was available at all times. The feed composition of the diet and its nutrient concentrations are presented in Table S1.

When goats were 6 months old (mean ± SE, 188 ± 0.4 days), the body weights of all young goats were recorded, and jugular blood was sampled before morning feeding. Individual ADG was calculated as the difference between 6-month body weight and birth weight divided by the number of days. Jugular blood samples were centrifuged at 3,000 × *g* at 4°C for 10 min to obtain serum samples. All serum samples were quickly frozen in liquid nitrogen and stored at −80°C for further analysis.

Rectal feces were taken from all young goats (mean ± SE, 188 ± 0.4 days), and sample collection was programmed over 3 days in 3-h intervals so that all 24 samples represented every hour of a 24-h feeding cycle. Samples were immediately snap-frozen in liquid nitrogen. At the end of the sampling period, all 24 samples for each goat were pooled, mixed, and homogenized using a sterile slap homogenizer. Next, about 1 g of subsample was taken and stored at −80°C for metagenomic DNA extraction. The remainder of the sample was stored at −20°C for VFA and NH_3_-N analysis.

One week before sampling (mean ± SE, 175 ± 0.4 days), all goats were housed individually and allowed 1 week to acclimate to DMI measurement. The intake of all goats fed *ad libitum* was adjusted to allow for 10% daily leftovers. After the acclimation period, initial and remaining amounts of feed were weighed daily for 1 week. Feed samples were dried at 55°C for 48 h to obtain dry matter (DM) content of the ration. Daily DMI per goat was calculated by multiplying daily as-fed intake by the DM content of the ration.

### Determination of VFA and NH_3_-N in fecal samples.

The fecal VFA was determined according to protocols previously described by Li et al. (45). In brief, each 0.5-g subsample of thawed feces was mixed with 1 mL distilled water and centrifuged at 13,500 × *g* at 4°C for 10 min. A 2-mL volume of supernatant was mixed with 500 μL of metaphosphoric acid (250 g/L) and then centrifuged at 10,000 × *g* at 4°C for 15 min. One mL of the supernatant was mixed with 200 μL of crotonic acid (10 g/L) and then filtered through a 0.45-μm organic filter. The VFA in the filtrate were separated and quantified by gas chromatography (Agilent Technologies 7820A GC system; Agilent Technologies, Santa Clara, CA, USA) with a flame ionization detector and a fused silica column (AE-FFAP, 30 m × 0.25 mm × 0.33 μm; Agilent Technologies). The concentration of NH_3_-N in fecal supernatant was quantified using a SKALAR San++ continuous flow analyzer (Skalar, Breda, Netherlands) as described by Xue et al. (46).

### Measurement of serum parameters.

The concentrations of serum IgG were determined using a goat IgG ELISA Quantitation kit (Bethyl Laboratories, TX, USA) according to the manufacturer’s protocol as described by Morales-de la Nuez et al. (47). The concentrations of serum glucose (48) and urea nitrogen (49) were analyzed using the respective commercial kits (Jiancheng Bioengineering Institute, Nanjing, China) according to the manufacturer’s protocols. Serum TP, Alb, Glob, total cholesterol, triglycerides, HDL-C, and LDL-C were measured using a Cobas c311 automatic biochemical analyzer (Roche, Basel, Switzerland).

### Microbial DNA extraction and 16S rRNA gene sequencing.

Total DNA in fecal samples was extracted using the QIAamp DNA Stool minikit (Qiagen, Dusseldorf, Germany) according to the manufacturer’s protocol. The quality of DNA was visually checked by 1% agarose gel electrophoresis and the DNA concentration was determined at a 260:280 nm ratio (>1.8) using a NanoDrop 2000 spectrophotometer (Thermo Fisher Scientific, Waltham, MA, USA). The amplicon library was constructed by PCR amplification of the V3-to-V4 hypervariable region in bacterial 16S rRNA gene using the forward primer 338F (5′-ACTCCTACGGGAGGCAGCAG-3′) and the reverse primer 806R (5′-GGACTACHVGGGTWTCTAAT-3′), with the reverse primer containing a 6-bp barcode. The PCR products were separated by 2% agarose gel electrophoresis and then purified using an AxyPrep DNA Gel Extraction kit (Axygen Biosciences, Union City, CA, USA). The libraries were then pooled in equimolar concentrations and subjected to paired-end (PE300) sequencing on an Illumina MiSeq platform (Illumina, San Diego, CA, USA) according to standard protocols.

### Illumina sequencing data analysis.

Paired-end reads were assigned to samples based on their unique barcode and truncated by cutting off the barcode and primer sequence. The raw read pairs were overlapped and merged using FLASH (v1.2.11) (50). Quality filtering was performed on the raw reads to obtain high-quality clean tags according to fastp (v0.19.6) (51). Next, the data were imported into QIIME 2 v2021.8 for demultiplexing (52), and bacterial amplicon sequence variants (ASV) were obtained using DADA2 (53). According to the SILVA database (version 138), the ASV in each sample was assigned to the corresponding taxonomies at phylum, class, order, family, and genus, respectively (54). The microbial α-diversity was identified by calculating the Sobs, ACE, Chao1, Simpson, and Shannon indices using QIIME 2. The microbial β-diversity was determined by distance matrices generated from Bray-Curtis analysis, PCoA, and ANOMIS analysis. Microbial functions were predicted using Phylogenetic Investigation of Communities by Reconstruction of Unobserved States 2 (PICRUSt2) (55), and the identified functional features were determined using the KEGG database.

### Enterotype clustering.

The ASV-level relative abundance profiles of each sample were involved in enterotype analysis using Jensen-Shannon divergence and partitioning around medoid clustering (9, 10). The optimal clustering effect was evaluated by the Calinski-Harabasz index. PCoA was performed to visualize microbial clusters in all samples.

### Statistical analysis.

All goats were separated into two microbial clusters according to their enterotypes. A *t* test for independent samples was used for variables, including ADG, DMI, serum parameters, fecal VFA, and NH_3_-N. Differential taxonomies, α-diversity, and predicted functions were compared using a Wilcoxon’ rank-sum test between the two enterotypes, and the *P* value was corrected by false-discovery rate adjustment.

Correlations of gut microbiota with ADG, fecal VFA, and serum parameters were tested by Spearman’s rank correlation test, and *P* < 0.05 was considered significant. To construct microbial co-occurrence networks in two enterotypes, genera which had an abundance greater than 0.1% and were present in more than half of all samples were included in our analysis. A significant Spearman’s rank correlation (*r* > |0.2|, *P < *0.05) between two bacterial genera was considered co-occurrence event. The network structure was visualized using Cytoscape v3.7.1. All data are expressed as means ± SE.

### Data availability.

The data sets supporting the results of this study are included within the article and its additional supplemental material. The raw sequence data were submitted to the NCBI Sequence Read Archive (SRA) under accession no. PRJNA871392.
