# Supplementary material for: Microbial Enterotypes Shape the Divergence in Gut Fermentation, Host Metabolism, and Growth Rate of Young Goats
Source: Microbiol Spectr. 2023 Jan 10;11(1):e04818-22. doi: 10.1128/spectrum.04818-22 (PMC9927581; doi:10.1128/spectrum.04818-22)
Supplement: Supplemental file 1 — Supplemental material. Download spectrum.04818-22-s0001.pdf, PDF file, 0.5 MB [file spectrum.04818-22-s0001.pdf]

Supplementary tables

Table S1. Feed ingredient and chemical composition of the young goats

Ingredients, % of DM	Content	Chemical composition	Content
Alfalfa hay	39.90	DM, %	57.40
Corn silage	20.60	CP, % of DM	15.56
Corn	20.90	Starch, % of DM	21.68
Wheat bran	10.70	NDF, % of DM	30.35
Soybean meal	5.90	ADF, % of DM	15.61
$NaHCO_3$	0.30		
$CaHPO_4$	0.30		
NaCl	1.30		
Premix[1]	0.10		

[1]Premix contained (per kg) the following: Cu, 2,925 mg; Fe, 3,900 mg; Zn, 2,750 mg; Mn, 800 mg; vitamin A, 1,500 kIU; vitamin D_3, 500 kIU; and vitamin E, 5,500 IU.

DM = dry matter; CP = crude protein; NDF = neutral detergent fiber; ADF = acid detergent fiber.

Table S2. Differential microbiota analysis of gut microbiota in two enterotypes at genus level (%, relative abundance > 0.1%)[1]

Taxa	cluster1	cluster2	*P* value
Rikenellaceae_RC9_gut_group	4.18 ± 0.50	7.96 ± 0.47	< 0.001
Christensenellaceae_R-7_group	3.17 ± 0.30	8.23 ± 0.44	< 0.001
Bacteroides	2.49 ± 0.24	4.82 ± 0.33	< 0.001
norank_f_Muribaculaceae	4.72 ± 0.83	1.78 ± 0.24	< 0.001
Succinivibrio	3.84 ± 1.33	1.60 ± 0.46	0.030
Prevotella	3.32 ± 0.66	0.93 ± 0.20	< 0.001
Oscillospiraceae_NK4A214_group	1.02 ± 0.07	1.78 ± 0.12	< 0.001
norank_f_F082	0.84 ± 0.19	1.88 ± 0.28	< 0.001
Romboutsia	1.15 ± 0.22	1.44 ± 0.21	0.042
Monoglobus	1.05 ± 0.11	1.51 ± 0.10	< 0.001
Roseburia	1.91 ± 0.47	0.52 ± 0.08	< 0.001
Prevotellaceae_UCG-003	1.75 ± 0.45	0.78 ± 0.13	0.029
Clostridium_sensu_stricto_1	0.95 ± 0.18	1.37 ± 0.20	0.017
Prevotellaceae_NK3B31_group	2.08 ± 0.39	0.16 ± 0.07	< 0.001
unclassified_f_Prevotellaceae	1.48 ± 0.87	0.30 ± 0.07	0.024
Blautia	1.08 ± 0.13	0.40 ± 0.05	< 0.001
unclassified_f_Oscillospiraceae	0.54 ± 0.04	0.90 ± 0.07	< 0.001
Oscillibacter	0.91 ± 0.06	0.49 ± 0.05	< 0.001
Parabacteroides	1.26 ± 0.20	0.07 ± 0.02	< 0.001
norank_f_norank_o_Bacteroidales	0.45 ± 0.08	0.80 ± 0.07	< 0.001
Akkermansia	0.16 ± 0.06	1.07 ± 0.31	< 0.001
unclassified_c_Clostridia	0.40 ± 0.06	0.65 ± 0.05	< 0.001
Candidatus_Saccharimonas	0.62 ± 0.12	0.29 ± 0.05	0.011
Faecalibacterium	0.89 ± 0.28	0.01 ± 0.00	< 0.001
norank_f_p-251-o5	0.29 ± 0.11	0.51 ± 0.10	< 0.001
Butyricicoccaceae_UCG-009	0.28 ± 0.02	0.55 ± 0.04	< 0.001
Anaerovoracaceae_Family_XIII_AD3011_group	0.28 ± 0.03	0.51 ± 0.05	< 0.001
unclassified_f_Ruminococcaceae	0.42 ± 0.06	0.27 ± 0.04	0.015
Prevotellaceae_UCG-004	0.07 ± 0.04	0.60 ± 0.12	< 0.001
Frisingicoccus	0.27 ± 0.07	0.30 ± 0.03	0.021
Rikenellaceae_dgA-11_gut_group	0.14 ± 0.02	0.37 ± 0.04	< 0.001
unclassified_f_Hungateiclostridiaceae	0.07 ± 0.03	0.34 ± 0.11	< 0.001
unclassified_o_Oscillospirales	0.15 ± 0.03	0.23 ± 0.03	0.003
Lachnospiraceae_UCG-010	0.16 ± 0.02	0.23 ± 0.02	0.012
Ruminococcaceae_CAG-352	0.33 ± 0.14	0.04 ± 0.01	< 0.001
unclassified_f_Peptostreptococcaceae	0.11 ± 0.02	0.26 ± 0.05	0.002

Candidatus_Soleaferrea	0.15 ± 0.01	0.20 ± 0.01	0.011
Colidextribacter	0.21 ± 0.03	0.12 ± 0.04	< 0.001
norank_f_Peptococcaceae	0.09 ± 0.01	0.20 ± 0.02	< 0.001
norank_f_norank_o_Rhodospirillales	0.19 ± 0.03	0.10 ± 0.01	0.022
norank_f_Barnesiellaceae	0.10 ± 0.02	0.18 ± 0.03	0.005
Dorea	0.11 ± 0.02	0.16 ± 0.02	0.027
Aeriscardovia	0.17 ± 0.03	0.11 ± 0.03	0.018
Eubacterium_brachy_group	0.08 ± 0.01	0.16 ± 0.01	< 0.001
norank_f_norank_o_norank_c_Clostridia	0.09 ± 0.01	0.13 ± 0.02	0.049

[1]Data were represented by mean ± SE. Cluster 1 (n = 39). Cluster 2 (n = 37).

Table S3. Microbial function prediction in two enterotypes (%, relative abundance > 0.1%)[1]

KEGG pathways	cluster 1	cluster 2	*P*-value
Biosynthesis of secondary metabolites	9.189 ± 0.013	9.137 ± 0.015	0.002
Biosynthesis of amino acids	4.470 ± 0.022	4.416 ± 0.016	0.015
Microbial metabolism in diverse environments	4.268 ± 0.009	4.326 ± 0.010	< 0.001
Carbon metabolism	2.809 ± 0.009	2.849 ± 0.008	0.001
ABC transporters	2.164 ± 0.027	2.085 ± 0.027	0.013
Amino sugar and nucleotide sugar metabolism	1.132 ± 0.009	1.099 ± 0.004	0.014
Oxidative phosphorylation	1.005 ± 0.008	1.035 ± 0.006	0.001
Carbon fixation pathways in prokaryotes	0.980 ± 0.007	1.006 ± 0.006	0.001
Alanine, aspartate and glutamate metabolism	0.975 ± 0.005	0.953 ± 0.003	< 0.001
Glycine, serine and threonine metabolism	0.973 ± 0.004	0.981 ± 0.003	0.024
Starch and sucrose metabolism	0.962 ± 0.015	0.905 ± 0.008	0.001
Peptidoglycan biosynthesis	0.945 ± 0.006	0.924 ± 0.003	0.010
Phenylalanine, tyrosine and tryptophan biosynthesis	0.856 ± 0.004	0.845 ± 0.004	0.019
Mismatch repair	0.847 ± 0.005	0.851 ± 0.003	0.008
Methane metabolism	0.739 ± 0.003	0.725 ± 0.002	< 0.001
DNA replication	0.697 ± 0.005	0.701 ± 0.003	0.004
Glyoxylate and dicarboxylate metabolism	0.691 ± 0.006	0.709 ± 0.003	0.007
RNA degradation	0.642 ± 0.002	0.654 ± 0.003	< 0.001
Galactose metabolism	0.638 ± 0.009	0.608 ± 0.005	0.007
Lysine biosynthesis	0.620 ± 0.002	0.609 ± 0.002	< 0.001
Citrate cycle (TCA cycle)	0.616 ± 0.006	0.648 ± 0.005	< 0.001
Butanoate metabolism	0.614 ± 0.005	0.628 ± 0.003	0.024
Flagellar assembly	0.611 ± 0.020	0.656 ± 0.013	0.044
Carbon fixation in photosynthetic organisms	0.547 ± 0.003	0.536 ± 0.001	< 0.001
One carbon pool by folate	0.542 ± 0.004	0.526 ± 0.003	0.001
Nicotinate and nicotinamide metabolism	0.472 ± 0.003	0.463 ± 0.001	0.019
Histidine metabolism	0.460 ± 0.004	0.452 ± 0.003	0.017
Arginine and proline metabolism	0.352 ± 0.004	0.366 ± 0.002	0.004
Vancomycin resistance	0.317 ± 0.002	0.309 ± 0.002	0.005
Biofilm formation - Escherichia coli	0.312 ± 0.005	0.301 ± 0.004	0.001
HIF-1 signaling pathway	0.284 ± 0.002	0.290 ± 0.001	0.014
Sulfur metabolism	0.253 ± 0.002	0.266 ± 0.003	< 0.001
Monobactam biosynthesis	0.247 ± 0.001	0.254 ± 0.001	< 0.001
Valine, leucine and isoleucine degradation	0.225 ± 0.004	0.252 ± 0.003	< 0.001
Cyanoamino acid metabolism	0.223 ± 0.003	0.210 ± 0.001	0.001
Antifolate resistance	0.213 ± 0.002	0.203 ± 0.001	< 0.001

Central carbon metabolism in cancer	0.206 ± 0.002	0.212 ± 0.001	0.003
Necroptosis	0.195 ± 0.001	0.189 ± 0.001	< 0.001
Longevity regulating pathway - worm	0.187 ± 0.001	0.190 ± 0.001	0.010
Fatty acid degradation	0.160 ± 0.002	0.169 ± 0.001	0.001
Novobiocin biosynthesis	0.142 ± 0.002	0.139 ± 0.001	0.041
Sphingolipid metabolism	0.132 ± 0.003	0.122 ± 0.002	0.027
Insulin resistance	0.118 ± 0.001	0.116 ± 0.001	0.039
AMPK signaling pathway	0.110 ± 0.002	0.116 ± 0.001	0.022
Tryptophan metabolism	0.109 ± 0.002	0.123 ± 0.002	< 0.001

[1]Data were represented by mean ± SE. Cluster 1 (n = 39). Cluster 2 (n = 37).

Supplementary figure

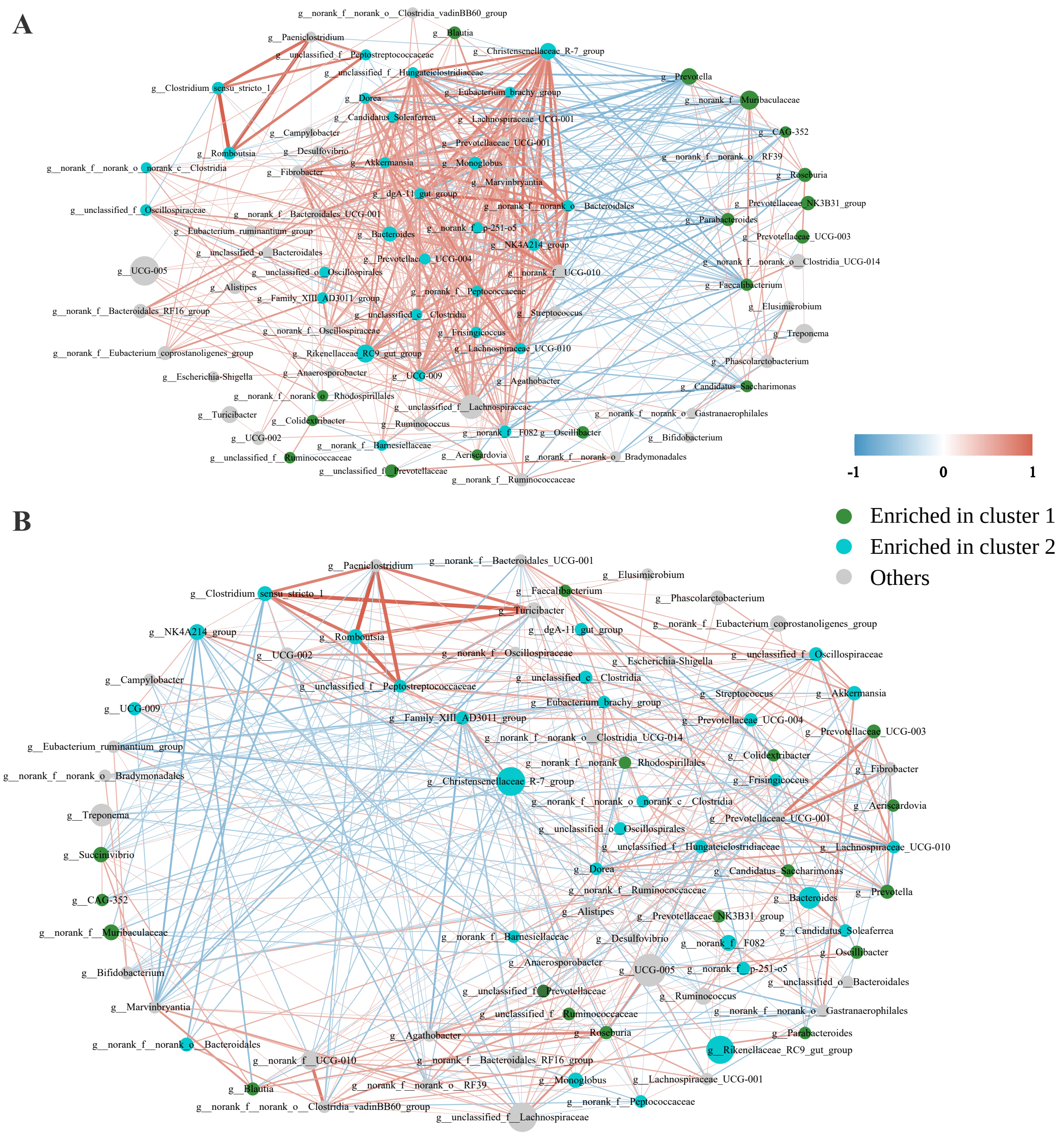

Fig. S1 Co-occurrence network of fecal microbial genera (relative abundance > 0.1%, detected in > 50% of all samples) in young goats with different enterotypes. (A) Co-occurrence network of gut microbial genera in cluster 1. (B) Co-occurrence network of gut microbial genera in cluster 2. Red lines represent positive correlations and blue lines represent negative correlations. Thickness of lines represents strength of relatedness. The colors of nodes indicate the clusters in which the genera enriched.
